# Impact of Phylogenetic Tree Completeness and Mis-specification of Sampling Fractions on Trait Dependent Diversification Models

**DOI:** 10.1093/sysbio/syad001

**Published:** 2023-01-16

**Authors:** Poppy Mynard, Adam C Algar, Lesley T Lancaster, Greta Bocedi, Fahri Fahri, Cécile Gubry-Rangin, Pungki Lupiyaningdyah, Meis Nangoy, Owen G Osborne, Alexander S T Papadopulos, I Made Sudiana, Berry Juliandi, Justin M J Travis, Leonel Herrera-Alsina

**Affiliations:** School of Biological Sciences, University of Aberdeen, Aberdeen, UK; Department of Biology, Lakehead University, Thunder Bay, ON, Canada; School of Biological Sciences, University of Aberdeen, Aberdeen, UK; School of Biological Sciences, University of Aberdeen, Aberdeen, UK; Department of Biology, Tadulako University, Palu, Indonesia; School of Biological Sciences, University of Aberdeen, Aberdeen, UK; Museum Zoologicum Bogoriense, Research Center for Biology, Indonesian Institute of Sciences, Cibinong 16911, Indonesia; Faculty of Animal Husbandry, Sam Ratulangi University, Kampus Bahu Street, Manado 95115, Indonesia; School of Natural Sciences, Bangor University, Environment Centre Wales, Deiniol Road, Bangor LL57 2UW, UK; School of Natural Sciences, Bangor University, Environment Centre Wales, Deiniol Road, Bangor LL57 2UW, UK; Microbial Ecology Research Group, Research Center for Biology, Indonesian Institute of Sciences, Cibinong 19611, Indonesia; Department of Biology, Faculty of Mathematics and Natural Sciences, IPB University, Bogor, 16680, Indonesia; School of Biological Sciences, University of Aberdeen, Aberdeen, UK; School of Biological Sciences, University of Aberdeen, Aberdeen, UK

## Abstract

Understanding the origins of diversity and the factors that drive some clades to be more diverse than others are important issues in evolutionary biology. Sophisticated SSE (state-dependent speciation and extinction) models provide insights into the association between diversification rates and the evolution of a trait. The empirical data used in SSE models and other methods is normally imperfect, yet little is known about how this can affect these models. Here, we evaluate the impact of common phylogenetic issues on inferences drawn from SSE models. Using simulated phylogenetic trees and trait information, we fitted SSE models to determine the effects of sampling fraction (phylogenetic tree completeness) and sampling fraction mis-specification on model selection and parameter estimation (speciation, extinction, and transition rates) under two sampling regimes (random and taxonomically biased). As expected, we found that both model selection and parameter estimate accuracies are reduced at lower sampling fractions (i.e., low tree completeness). Furthermore, when sampling of the tree is imbalanced across sub-clades and tree completeness is ≤ 60%, rates of false positives increase and parameter estimates are less accurate, compared to when sampling is random. Thus, when applying SSE methods to empirical datasets, there are increased risks of false inferences of trait dependent diversification when some sub-clades are heavily under-sampled. Mis-specifying the sampling fraction severely affected the accuracy of parameter estimates: parameter values were over-estimated when the sampling fraction was specified as lower than its true value, and under-estimated when the sampling fraction was specified as higher than its true value. Our results suggest that it is better to cautiously under-estimate sampling efforts, as false positives increased when the sampling fraction was over-estimated. We encourage SSE studies where the sampling fraction can be reasonably estimated and provide recommended best practices for SSE modeling. [Trait dependent diversification; SSE models; phylogenetic tree completeness; sampling fraction.]

Understanding the origins of diversity and why some clades are more diverse than others are fundamental questions in evolutionary biology. Estimating the diversification rate of lineages, and how they vary across phylogenetic trees, is essential to developing this understanding. The per-lineage rates of speciation and extinction in a clade can be affected by environmental and geographical factors (Ricklefs 2007), time (or clade age; Henao Diaz et al. 2019), and species’ traits (Jablonski 2008; Rabosky and McCune 2010). Possessing a certain trait state can promote speciation by increasing fitness or reproductive output (Liem 1973; Weber and Agrawal 2014; Helmstetter et al. 2016; Laenen et al. 2016; Igea et al. 2017). Conversely, some trait states may hinder speciation or increase extinction rates; for example, specialization has been shown to reduce speciation rates in some clades (Day et al. 2016), but promotes it in others (Resl et al. 2018; Tonini et al. 2020). Although differences in diversification rates across trait states occur frequently (Jablonski 2008), it does not necessarily mean that the trait itself drives alone the clades’ diversification dynamics.

The state-dependent speciation and extinction (SSE) framework was developed to determine the impact that the evolution of a trait has on subsequent patterns of lineage diversification through time, by linking the presence/absence (or value) of trait states to diversification rates. To implement this approach and determine the most likely mode of diversification, Examined Trait Dependent (ETD) models, which include the trait hypothesized to affect diversification, are compared to models with Constant Diversification Rates (CR models) and Concealed Trait Dependent (CTD) models [also known as Character-Independent (CID) models] (Beaulieu and O’Meara 2016). CTD models account for the possibility that diversification rates do not vary in relation to the focal trait, but rather with some unmeasured trait(s). The CTD model is always as complex (in terms of the number of character states) as the ETD model (in CID notation, the number of character states has to be specified for example, CID-2 has two character states). The use of CTD models successfully reduces false inferences of trait dependent diversification that were problematic in earlier SSE tools (Rabosky and Goldberg 2015; Beaulieu and O’Meara 2016) such as BiSSE (Maddison et al. 2007a) and QuaSSE (Fitzjohn 2010). The improved suit of SSE models includes HiSSE (hidden-state-dependent speciation and extinction) (Beaulieu and O’Meara 2016), GeoHiSSE (a biogeographical version) (Caetano et al. 2018), MuHiSSE (a multi-trait state version) (Nakov et al. 2019; Zenil-Ferguson et al. 2019), MiSSE (Vasconcelos et al. 2022), and SecSSE (several examined and concealed state dependant speciation and extinction) (Herrera-Alsina et al. 2019).

The accuracy of model selection (i.e., comparing ETD, CTD and CR models) and parameter estimation (speciation, extinction, and transition rate estimates) in state-dependent diversification analyses is dependent on several factors, including: the similarity of true speciation rates across trait states, the number of transitions among trait states, completeness of trait information, and accuracy and completeness of the phylogenetic tree. It is harder to find evidence for trait dependent diversification when speciation rates are similar to each other (Beaulieu and O’Meara 2016) and when extinction rates are high (Herrera-Alsina et al. 2019). Incomplete trait information adds uncertainty to models: a missing tip in the phylogenetic tree or lack of trait information for an extant species will render the contribution of that branch to the analysis null. Some tools, such as SecSSE, can account for partial trait state data which reduces the negative effect of missing trait information (Herrera-Alsina et al. 2019). Missing tips can result in loss of trait state transitions; this is a crucial piece of information for SSE modeling and lost transitions will likely negatively impact the analysis. Moreover, phylogenetic trees are sources of uncertainty themselves, with potential inaccuracies both in the topology of the tree and the branch lengths (Felsenstein 1985; Donoghue and Ackerly 1996).

Diversification analyses are normally intended for complete clades, but phylogenetic trees may be incompletely sampled, or only a (potentially polyphyletic) subset of taxa may be chosen for analysis. For example, the number of species/phylotypes in the entire clade may be unknown (e.g., in microbes) or the taxonomic scope of a study could be geographically limited. In SSE models, the sampling fraction is the percentage of taxa in the clade included in the phylogenetic tree (Nee et al. 1994; Fitzjohn et al. 2009; Chang et al. 2020), is specified separately in each trait state. The sampling fraction setting typically assumes that taxa are missing from the clade at random; however, this is rarely true and does not reflect realistic sampling associated with empirical phylogenies. Older, more abundant lineages are less likely to be excluded than younger lineages in incompletely sampled trees (Davies et al. 2011). In some studies, certain sub-clades, for example those from the tropics in contrast to temperate counterparts, may be highly under-sampled or completely excluded, potentially affecting the accuracy of SSE analysis (Titley et al. 2017). In studies that focus on a specific geographic region, species from outside that region would be removed. Plant collection biases have been well documented (Rich and Woodruff 1992; Moerman and Estabrook 2006; Corlett 2016; Daru et al. 2018) and some families (e.g., Asteraceae, Cyperaceae, and Poaceae) have phylogenetic biases in collection frequency (Daru et al. 2018), so sampling is neither uniform nor random across these phylogenies. Animals that are particularly prone to taxonomic bias include invertebrates such as Insecta, Arachnida, and Gastropoda (Troudet et al. 2017). Larger animals are not exempt from biases; for example, there is a lack of genetic data for bird species from tropical regions (Reddy 2014). Indeed, there is a general pattern of under-sampling of tropical species compared to those in temperate areas (Chek et al. 2003; Collen et al. 2008; Titley et al. 2017). Microbial phylogenetic reconstructions are likely to almost always be under-sampled and there may often be biases related to geography or environmental conditions, for example with higher sampling occurring under certain pH conditions (Gubry-Rangin et al. 2015).

In cases where the total number of species in a clade is unknown, the sampling fraction could be mis-specified, and this too may affect diversification analyses. Issues with clade specific sampling factions have previously been documented (Beaulieu 2020) but little work has been done on the effects of sampling extent, tree imbalance, and mis-specification on SSE model accuracy (Nee et al. 1994; Fitzjohn et al. 2009; Chang et al. 2020). However, understanding the consequences of different sampling regimes on model comparison and parameter estimation is paramount to give confidence to SSE studies with incomplete phylogenetic trees. Conducting SSE analyses in a Bayesian framework may be a useful method to reduce uncertainty around parameter estimation and improve the ability to detect signals of trait-dependence diversification. For instance, with Bayesian analysis is possible to account for sampling fraction uncertainty, by providing a range of possible sampling fractions as a prior, although this is not something that has been implemented in studies yet.

Here, we provide the first in-depth evaluation of how incomplete phylogenetic information affects the performance of SSE models that incorporate concealed/hidden traits. We simulated data sets (phylogenetic trees along with trait information) and fitted SSE models in order to evaluate model selection under a number of different scenarios. Specifically, we evaluate the ability to select the correct type of relationship between trait evolution and branching patterns, and the accuracy in estimating parameter values (speciation, extinction, and transition rates). We focus on four key variables: 1) sampling fraction; 2) phylogenetic tree size; 3) sampling regime, in which phylogenies were sampled randomly or with taxonomic bias; and 4) mis-specification of sampling fraction (where the true clade size is either under- or over-specified). We also used Bayesian analysis with a prior on the sampling fraction to deal with unknown sampling fractions.

## Materials And Methods

### Trait and Phylogeny Simulation

**Figure 1.**
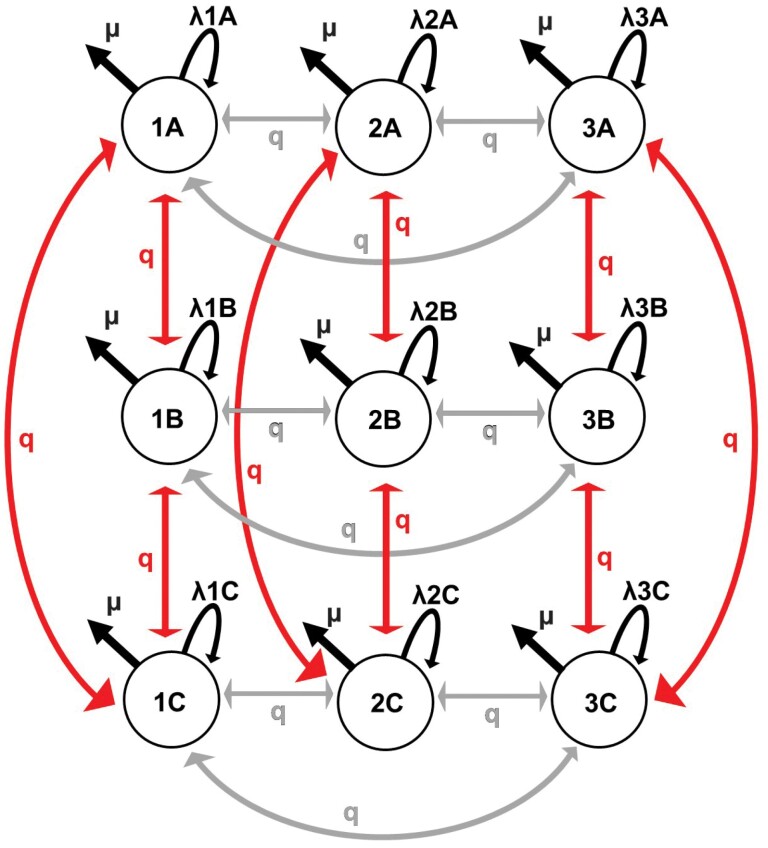
Schematic model diagram. Each circle indicates a trait state combination with one examined trait state (1, 2, or 3) and one concealed trait state (A, B, or C). The speciation rate (*λ*) for each trait state combination differed either with the examined trait (ETD model), or the concealed trait (CTD model). The extinction rate (*μ*) was the same in every trait state combination. The transition rates (*q*), shown in grey for the examined trait and red for the concealed trait, was symmetrical, that is, the same rate was used for every transition, and all transitions between trait states were possible.

We simulated phylogenetic trees and accompanying trait data under three types of trait dependent diversification—ETD, CTD, and Constant Rate (CR). We applied the same simulation procedure described in Herrera-Alsina et al. (2019). In a nutshell, the simulations are run in continuous time, where first the waiting times are taken from an exponential distribution whose parameter is the sum of the per-lineage rates of events (i.e., speciation, extinction, and trait evolution). Then, a species is randomly taken to undergo one of the events and a record of it is kept. Specifically, we simulated the evolution of two traits that were independent from one another. Each trait had three states (examined trait with states 1, 2 and 3; concealed trait with states A, B, and C, see below) and all transitions across the three states were possible at a single rate (*q*). This means that the shift from state 1 to state 2 had the same rate than shifting from state 2 to state 1. Notice that both traits are combined to yield a nine-state system (1A, 2A, 3A, 1B, 2B, 3B, 1C, 2C, 3C) where double transitions (e.g., the change from 1A to 2C) are not possible. Although more complex systems (traits states ≥ 4) are possible in SecSSE (Herrera-Alsina et al. 2019), we chose three to minimize complexity. One of the traits, the examined trait, influenced diversification such that every time a lineage switched to a different trait state, its speciation/extinction rate was adjusted accordingly. This trait was the only factor affecting speciation and extinction dynamics and its’ three states were numerically coded (i.e., 1, 2, 3). In contrast, the other trait, the concealed trait, did not influence diversification, but rather evolved neutrally over time. This trait’s states were denoted alphabetically (A, B, C). Because the concealed trait had the same number of states as the examined trait (Fig. 1), the CTD model used here is equivalent to the character-independent (CID-3) model in HiSSE (Beaulieu and O’Meara 2016; Herrera-Alsina et al. 2019). We kept track of the evolution of both traits and at the end of the simulation we retained the species’ trait states of either the examined trait (ETD-generating model) or the concealed trait (CTD-generating model). Note that the CTD model accounts for trait dependent diversification of an unmeasured trait (the concealed trait), and it is equal in complexity to the observed trait model. We also ran a simulation where both traits were neutral, and the rate of diversification did not change across time or lineages (CR-generating model). In the ETD-generating model, speciation rates differed only across examined trait states *λ*1A = *λ*1B = *λ*1C ≠ *λ*2A = *λ*2B = *λ*2C ≠ *λ*3A = *λ*3B = *λ*3C, while in the CTD-generating model, speciation rates differed only across concealed trait states *λ*1A = *λ*2A = *λ*3A ≠ *λ*1B = *λ*2B = *λ*3B ≠ *λ*1C = *λ*2C = *λ*3C (Fig. 1). In the CR-generating model, all speciation rates were the same regardless of trait state *λ*1A = *λ*1B = *λ*1C = *λ*2A = *λ*2B = *λ*2C = *λ*3A = *λ*3B = *λ*3C. The resulting simulated datasets included phylogenetic trees as well as the trait states associated with them.

We set the speciation and extinction rates within the ranges previously used to test the performance of SecSSE analyses (Herrera-Alsina et al. 2019). For ETD and CTD generated phylogenies, the speciation rates (*λ*) were: 0.1, 0.3, 0.5; for CR generated phylogenies, all trait states had the same speciation rate of 0.3. The extinction rate (*μ*) was set either low (0.001) or regular (0.05). We did not include models with variable extinction rates (and/or different transition rates) which adds to the model complexity and may lead to confounding effects (Davis et al. 2013). The transition rate (*q*) was set to 0.4 for all transitions in all phylogenies, and all transitions between the three states were possible (Fig. 1). The transition rate is somewhat high, although not unrealistic: while some empirical SSE studies have found transition rates similar to the rate we chose (e.g., pollination in hummingbirds, Wessinger et al. 2019), other studies have found lower rates [e.g., habitat preference in Diatoms Nakov et al. (2019); host-plant association in dragonflies (Letsch et al. 2016); body shape and habitat preference in marine fishes (Rincon-Sandoval et al. 2020)], or much higher rates [mycorrhizal association in fungi (Looney et al. 2016)]. This also enabled testing the performance of SecSSE under new conditions, as its performance has already been documented with transition rates of 0.05 and 0.1 (Herrera-Alsina et al. 2019). Phylogenetic trees were simulated in three size groups; as younger trees have fewer tips, this was achieved by altering the crown age of trees: large (1000–5000 tips; age = 23 MY), medium (450–650 tips; age = 19 MY) and small (100–250 tips; age = 13.4 MY). The range in number of species within each category of tree size is due to stochasticity and extinction rate (even with the same clade age). For each tree size, we simulated 100 phylogenies of each diversification mode (ETD, CTD, CR) with low extinction, giving a total of 300 trees per size group. For small and medium tree sizes, we also simulated 100 phylogenies of each diversification mode with regular extinction.

### Phylogenetic Tree Trimming

Phylogenetic trees and accompanying trait data were trimmed (removal of extant tips), either randomly or with a taxonomic bias (Supplementary Fig. S1), to generate five sampling fraction (SF) levels in 20% intervals, that is SF: 100% (the full tree), 80%, 60%, 40%, and 20%. For random trimming, tips were randomly removed from across the phylogenetic tree [which is how SSE models treat the SF of a phylogeny, with the SF specified for each trait state (Nee et al. 1994; Fitzjohn et al. 2009; Chang et al. 2020)]. To generate taxonomically biased sampling, we selected one or two sub-clades (containing 20–30% of the clade’s size) to be heavily trimmed (removal of 80–90 % of tips) (Supplementary Fig. S1). This resulted in slight variation in the final SF (± 2%) in each SF level, but this was less than the variation in trait state percentages (see below).

Randomly trimmed sets were created for all three phylogenetic tree sizes while taxonomic bias trimming was performed only on medium sized phylogenetic trees. Note that we did not explicitly evaluate the effect of a *trait* bias: tip loss was done agnostically with respect to underlying trait state distributions. High tip ratio bias (e.g., one trait state accounting for < 10% of tips) can reduce model power and accuracy of parameter estimates (Davis et al. 2013). We therefore checked for tip ratio bias and found that all trait states were trimmed to a similar percentage—each of the three trait states accounted for ~33% of tips (Supplementary Fig. S2) and there were no differences in trait state percentages across tree sizes or trimming methods. Although our transition rate (*q* = 0.4) guarantees that transitions events are distributed throughout time and lineages, unsampling some tree tips might lead or not to the loss of trait state transitions events. The type of trait state transitions lost during trimming may affect model power. Therefore, during simulation of the regular extinction rate phylogenetic trees, the trait state transitions were recorded; for those datasets where the inference fails to select the right model, we explored whether they feature asymmetric transition lost (e.g., more 1 -> 2 lost than 2 -> 1) or dissimilar prevalence of concealed/examined transitions (e.g., more A -> B lost than 1 -> 2).

### Sampling Fraction Settings in Maximum Likelihood (ML) Framework

Using the above sets of simulated trees and trait states at the tips, SSE model analyses were performed under two different scenarios: 1) with the SF correctly specified, and 2) with the SF incorrectly specified (see Table 1 for details). The SF was specified per trait state: for example, for SF 60%, the SF was specified as 0.6 for each of the three trait states (notice that in hisse there is only global SF that accounts for phylogenetic incompleteness). Additionally, we provided a narrow and a wide prior for SF in a Bayesian context (see below).

**Table 1:** Mis-specified SF sets

	Sampling fraction (%)				
True SF	100	80	60	40	20
Mis-specified SF	80	100 60	100 80 40	100 80	–

Note: 100% is a complete phylogenetic tree; 20% is a phylogenetic tree containing only 20% of tips from the complete tree. In mis-specified SF, columns indicate what the True SF was mis-specified as; for example, True SF 80%, was mis-specified as SF 100% and SF 60%. Mis-specified sets were done on random and bias trimmed medium sized trees.

### Evaluation of SSE Models in ML

To test the ability of SSE models to select the correct (generating) model of trait dependent diversification under the above scenarios, we ran SecSSE analyses under the three models (ETD, CTD and CR) and compared Akaike information criterion values (AICc) (Bekara et al. 2005) and Akaike Weights (Wagenmakers et al. 2004). The percentage of false positives (i.e., erroneous model selection of ETD diversification) is calculated from the number of cases where ETD was selected as the best model in CTD and CR generated datasets, divided by the total number of CTD and CR generated datasets. False negatives (i.e., erroneous rejection of ETD diversification) are the percentage of ETD generated datasets that had CTD or CR selected as the best model. For medium sized, regular extinction trees, that were ETD generated, we also tested an ECTD model, which is a combination of the ETD and CTD, equivalent to the MuHiSSE. If ECTD was selected as the best model, it would indicate that the trait of interest has some effect on diversification dynamics but is not solely responsible. We explored the robustness of the results in regard to tree characteristics (tree size and imbalance). The Sackin Index (Sackin 1972) was used as a measure of tree imbalance: it is the average path length from tree root to tip (Blum and François 2005), and the less balanced the tree, the larger its Sackin Index value.

### Evaluation of SSE Models in Bayesian Framework

We also explored model selection in the presence of incomplete tree sampling under a Bayesian framework. Bayesian implementations of SSE models are available in RevBayes (Höhna et al. 2014), mcmc-diversitree (Silvestro et al. 2014), and BEAST [the “SSE” package; (Mendes et al. 2018; Bouckaert et al. 2019)]. RevBayes is particularly flexible, enabling various priors to be set, for example on the root frequencies, and allows for uncertainty in the tree topology and branch lengths to be marginalized over. The scripts provided in Freyman and Höhna (2018) are intended to compare the likelihood of state-dependent and state-independent models using RevBayes (Höhna et al. 2016), which fits our purpose. We simulated 50 ETD datasets using the same procedure as in our main analysis (but considering only two examined and two concealed states, tree size ranged from 100 to 250 species; see note on computing time below), where 60% of the species were kept under both trimming methods: random and biased. We ran RevBayes’s routine for state-dependent and state-independent models on each simulated dataset with two different setups for the SF. In one setup, we used a uniform prior with lower bound of 0.3 and upper bound of 0.9 (i.e., wide prior), whereas the other setup featured lower bound of 0.5 and upper bound of 0.7 (i.e., narrow prior). We used a stepping-stone approach to compute the marginal likelihood and bayes factor (Kass and Raftery 1995) to find the model with the highest statistical support. The major computational resources necessary to conduct this analysis prevented us from testing other scenarios under Bayesian framework (for the RevBayes analysis we used 192000 hours of computing time: two sampling methods × two priors × two dependence modes × 50 trees, 20 days each).

## Results

### Effects of Phylogenetic Tree Size and Sampling Fraction, When the Sampling Fraction is Known and Taxonomically Unbiased

**Figure 2.**
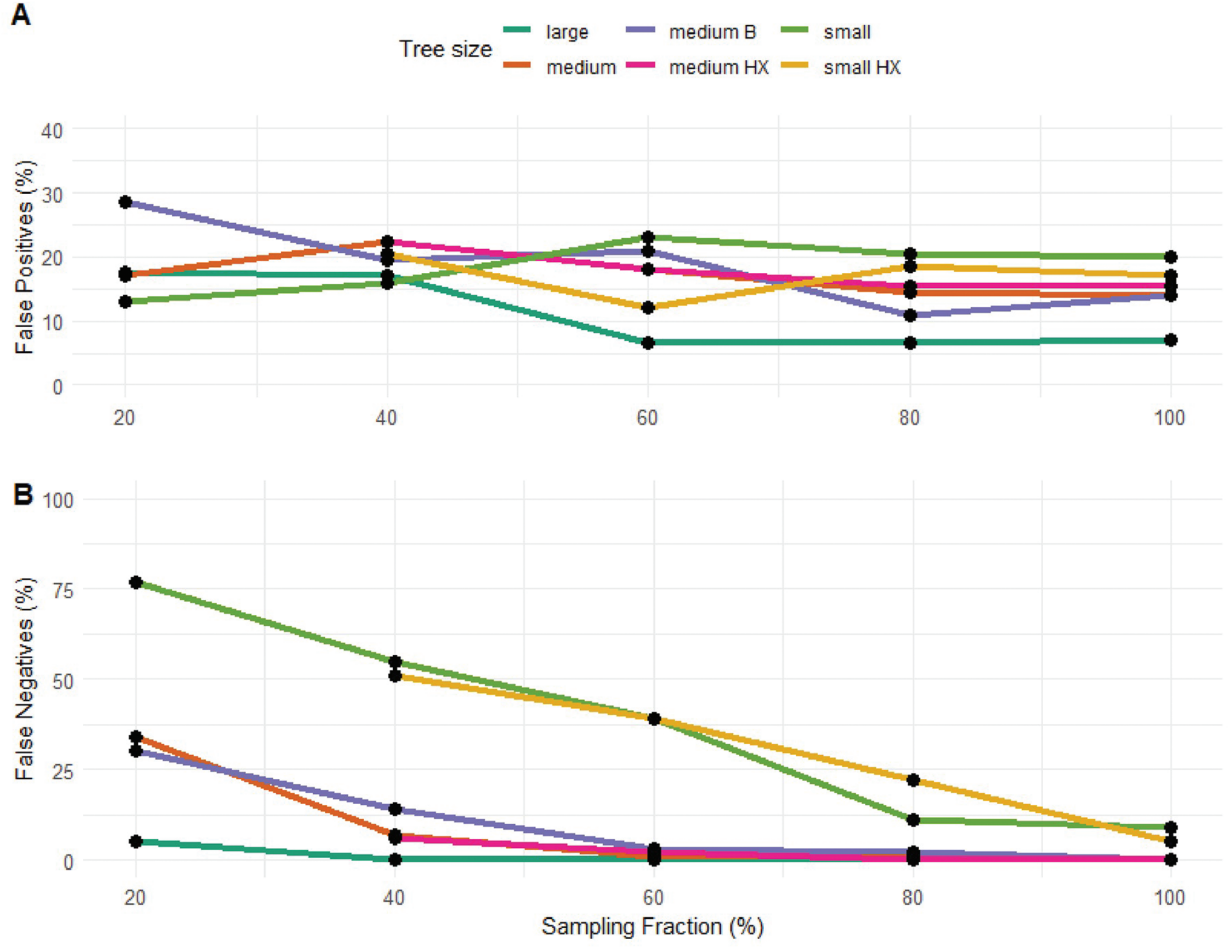
False positives (A) and false negatives (B) from all correctly specified sampling fraction sets, including randomly trimmed large, medium, and small trees, as well as bias trimmed trees (medium B), and trees with different extinction rates (small HX and medium HX).

To test the effect of phylogenetic tree size, we compared randomly trimmed phylogenies of large (1000–5000 tips), medium (450–650 tips), and small (150–250 tips) sizes. As expected, SecSSE performed best with larger trees and those with more complete sampling (Fig. 2; Supplementary Table S1). Correct model selection was reduced in smaller phylogenetic trees, and under low SFs across all phylogeny sizes. For large trees, the false positive rate was ~ 6.5% when the SF ≥ 60, and increased to 17% false positives rate at SF 40 (Supplementary Table S1, set 10; Fig 2). Medium sized trees had false positive rates of ~ 14.5% when SF ≥ 80, this rate became > 22% at SF 40. Small trees had a higher false positive rate of 20% at SF 100, this decreased to 16% at SF 40, however, the rates of false negatives increased dramatically: from 9% at SF 100, to 55% at SF 40 (Supplementary Table S1; Fig 2). In contrast, false negatives were negligible (< 5%) for large and medium sized trees when the SF was ≥ 60% (Fig. 2 and Supplementary Table S1). Results were very similar for trees with higher extinction rates (Fig.2 and Supplementary Table S3).

**Figure 3.**
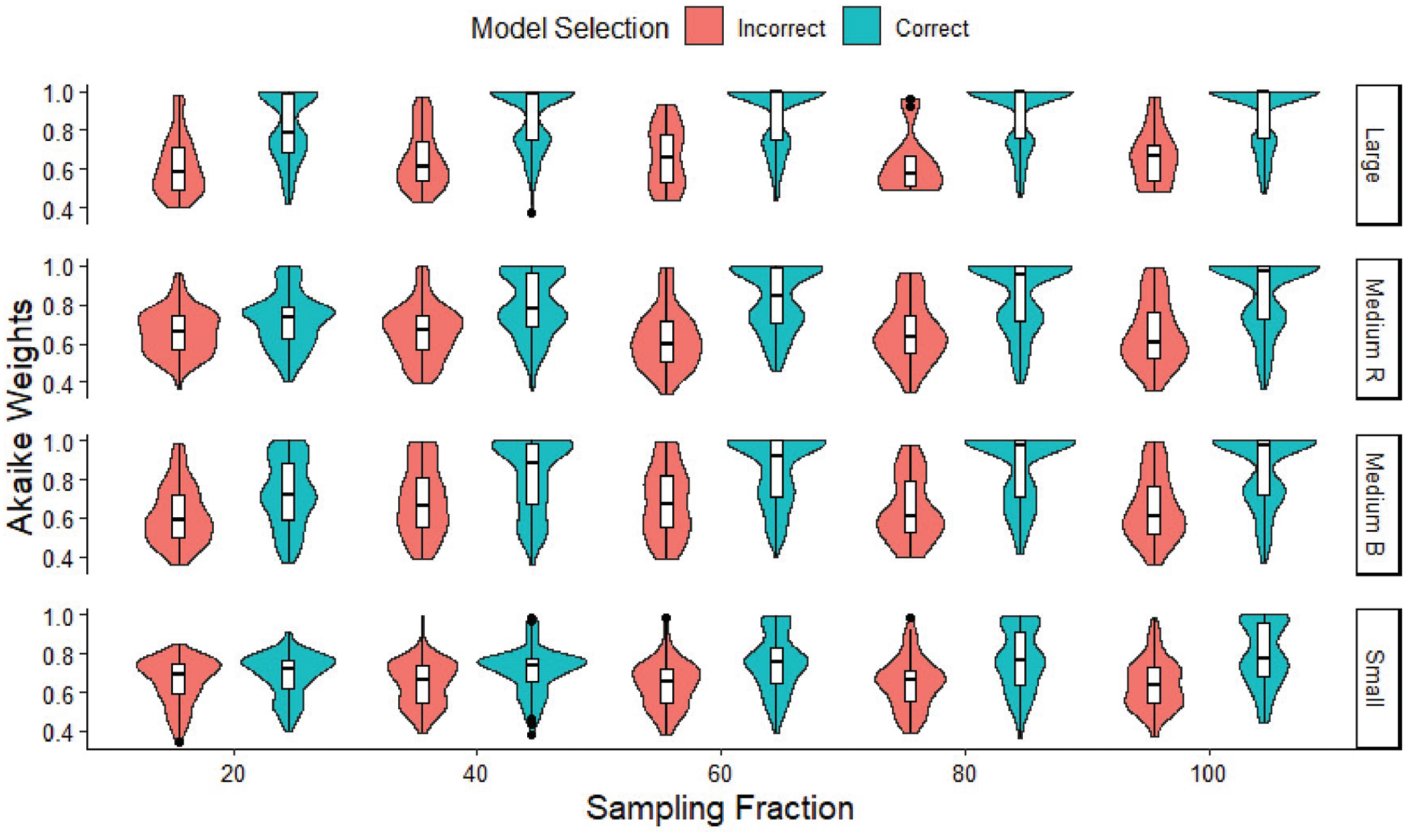
Model selection. Akaike weights of correctly (blue) and incorrectly (red) selected models of each size and trimming method (R = randomly trimmed; B = biased trimmed), at each SF. All three generating models for each SF are grouped into the violin plot; for each pair of violin plots *n* = 300.

Akaike weights were higher in correctly selected models compared to incorrectly selected models (Fig. 3); the difference was most pronounced in large trees, and less so in small trees. Mean Akaike weight values for correctly and incorrectly selected models were more similar at lower SFs (Fig. 3). The ECTD model was heavily penalized in the AICc analysis due to too many free parameters (11 in ECTD compared to 5 in ETD) and as such was never selected as the best model, even with ECTD simulated phylogenetic trees (Supplementary Table S4). With so many variables in the ECTD model, the parameter space becomes too wide, resulting in some model runs being incomplete even after 10 optimization cycles.

Tree imbalance, as determined via Sackin index, did not affect correct model selection for complete or randomly trimmed phylogenies The random sampling procedure removed tree tips randomly and consequently transition events were also removed randomly as we found that number of transitions out of a given state were not different than transitions going into that state (e.g., 1 -> 2 = 2 -> 1). Moreover, the number of transitions lost across the examined trait were similar than in the concealed trait (Supplementary Fig. S3).

**Figure 4.**
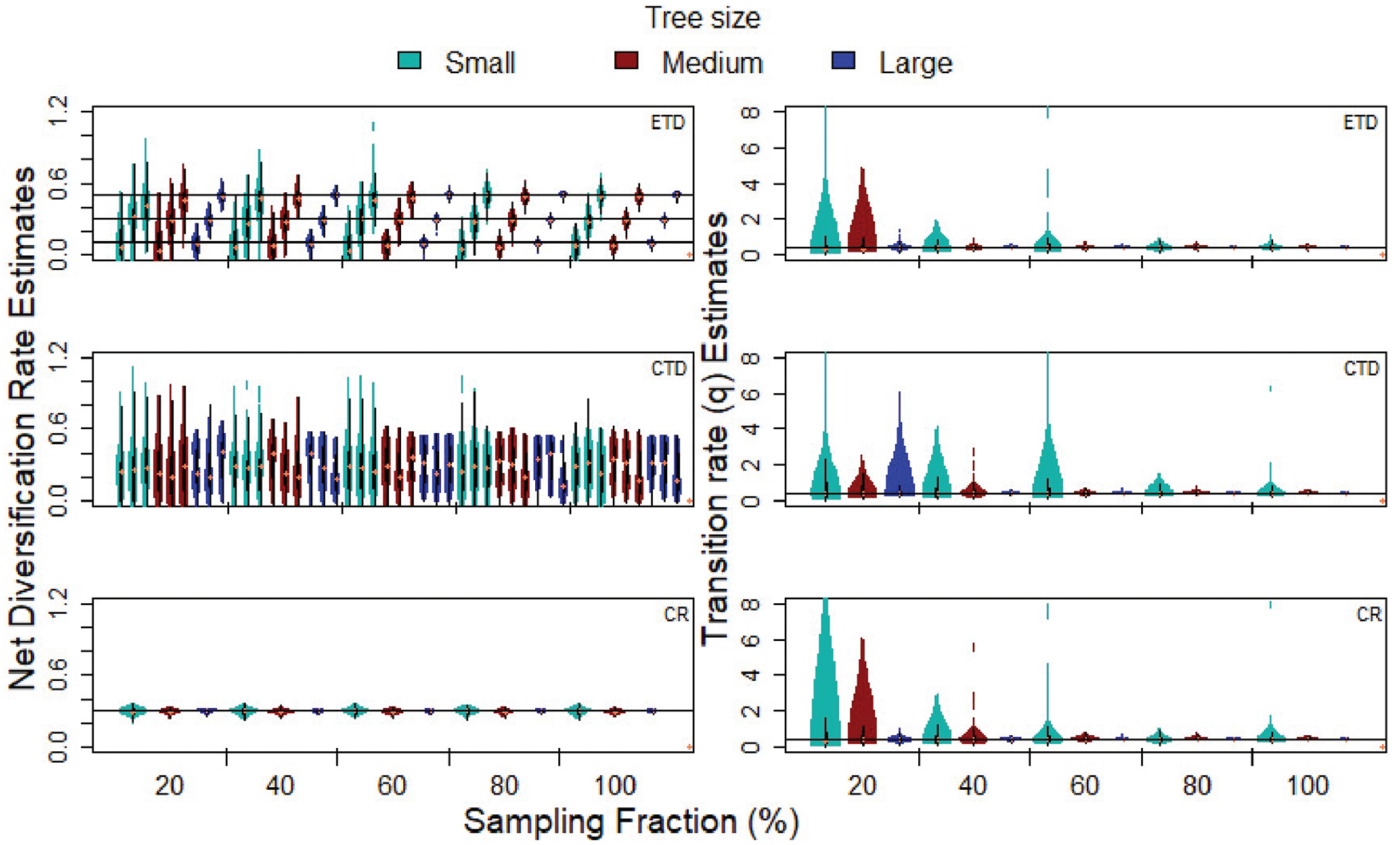
Parameter estimates for correctly specified SF sets of randomly trimmed ETD (examined trait dependent), CTD (concealed trait dependent) and CR (constant rate) generated trees. Colors indicate tree sizes. The horizontal line indicates the true parameter value. Note that these plots include all generated trees, regardless of whether the generating model was selected as the best model or not.

Parameter estimation was more accurate and precise at higher SFs, whereas variation in parameter estimates increased with decreasing SF (Fig. 4). Small trees had the largest variation in their parameter estimates, while large trees had the smallest variation (Fig. 4). The only exception was the net diversification rates of CTD generated trees, which showed little difference in parameter estimate variation across tree size (Fig. 4). In most cases, large trees at SF ≥ 40 had less variation in parameter estimates compared to medium sized trees at SF 100 (Fig. 4). Extinction rates for phylogenies generated with low extinction (*µ* = 0.001) were generally over-estimated (e.g., for medium trees at SF 100, mean = 0.0284 ± 0.0371; median = 0.0111), whereas in the regular extinction sets (*µ* = 0.05), extinction rates were marginally under-estimated (e.g., for medium trees at SF 100, mean = 0.0442 ± 0.0418; median = 0.0384). There were occasional high outliers for the transition rate estimates; this occurred with ETD, CTD and CR generated trees, but only affected medium sized trees at SF 20 and small trees at SF 60 or lower (Fig. 4).

### Effects of Sampling Bias When the Sampling Fraction is Correctly Specified

**Figure 5.**
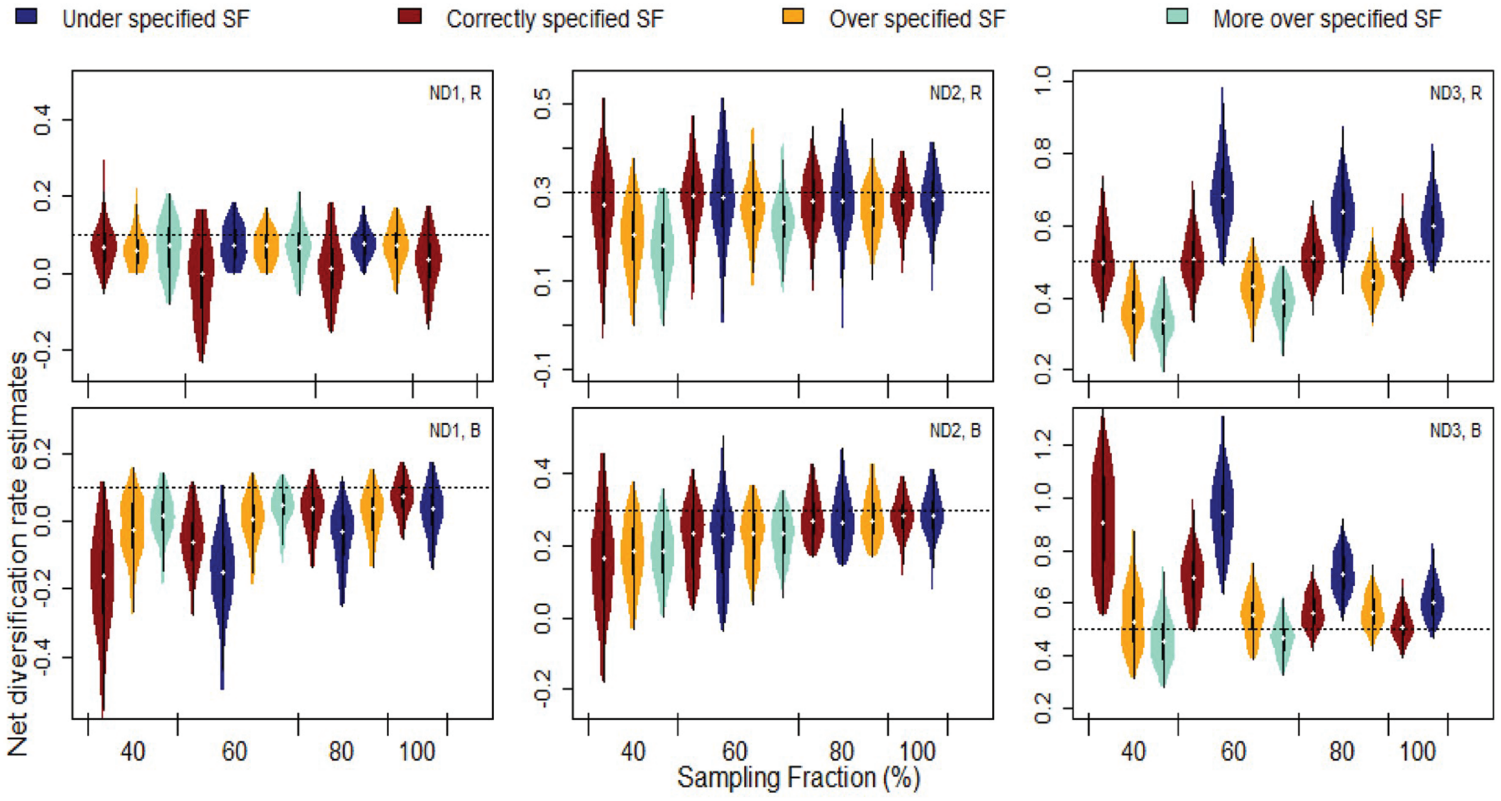
Net diversification rate estimates of the medium size ETD generated phylogenetic trees, that were randomly (R; top row) or biased (B; bottom row) trimmed. ND1, ND2 and ND3 indicate the net diversification rate for trait state 1, 2 and 3 respectively. Colors indicate if the SF was correctly specified or mis-specified. The horizontal line indicates the true parameter value. Plots include all generated trees for each set, regardless of whether the generating model was selected as the best model or not.

Medium sized phylogenetic trees that were trimmed randomly, or with taxonomic bias, were compared to test the effects of sampling bias. Random sampling led to better model performance than biased sampling for both model selection and parameter estimation (Supplementary Table S1, Figs. 2, and 4). Randomly trimmed trees had lower rates of false negatives than biased trimmed trees (Supplementary Table S1). Biased trimmed trees had a lower rate of false positives compared to randomly trimmed trees at SF 80, but at SF 60 this was reversed (Supplementary Table S1). At SF 20, the percentage of false positives was considerably higher for biased trimmed trees compared to randomly trimmed trees (Supplementary Table S1). In biased trimmed sets, most false positives came from an erroneous ETD selection of a CR generated tree (Supplementary Table S1). In contrast, for randomly trimmed trees, most false positives came from an erroneous ETD selection of a CTD generated tree (Supplementary Table S1). Net diversification rate estimates were considerably more accurate in randomly sampled trees (Fig. 5). For biased trimmed trees, as SF decreased, net diversification rate estimates became more inaccurate (Fig. 5). Figure 5 shows that, even though the rate estimates are not accurate, the model is able to detect differences across the three states and accommodates the rates of net diversification to maximize this difference.

### Effects of Mis-specifying the Sampling Fraction

**Figure 6.**
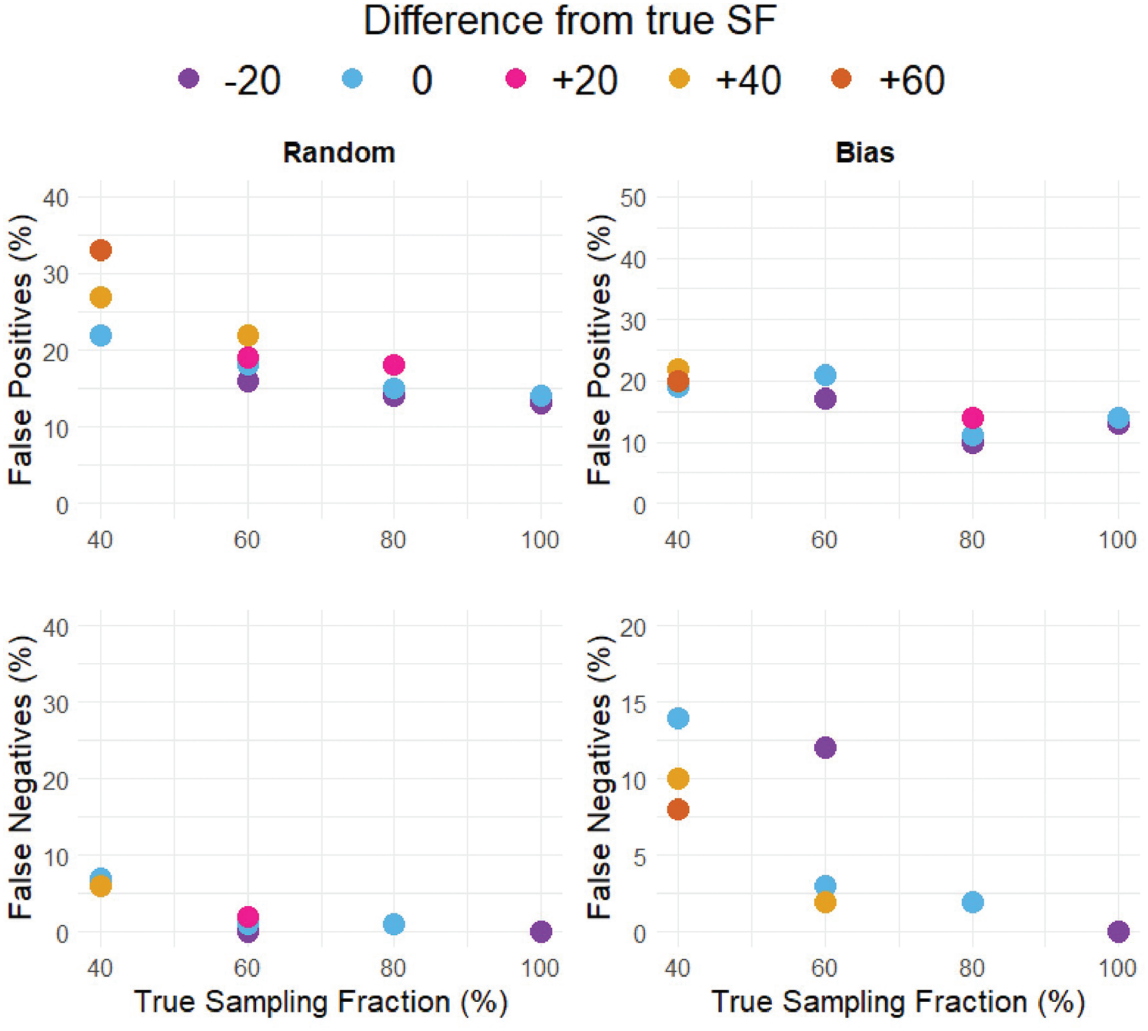
Percentages of false positives and false negatives in Random and Bias sampled sets. Colors indicated the difference from the true SF, that is if the true SF is 80, at −20 the SF was specified as 60, and at +20 the SF was specified as 100.

Mis-specifying the SF by ±20% (of the total number of tips) reduced the accuracy of model selection (Fig. 6) and parameter estimates (Fig. 5). Random and biased sampled phylogenetic trees were affected by mis-specification of the SF in a similar manner. Specifying the SF as higher than its true value often caused an increase in false positives (Fig. 6), while under-specifying the sampling faction gave similar or slightly lower rates of false positives compared to correctly specified SF sets (Fig. 4; Supplementary Table S2). Similar to correctly specified SF sets, false negatives were negligible (≤ 5%) when the true SF was ≥ 60%, irrespective of the degree of mis-specification; the only exception to this was Set 6b, which had a much higher false negative rate (Supplementary Table S2).

Mis-specifying the SF affected net diversification rate estimates in some sets, most noticeably “bias ETD”, “random ETD” and “random CR” (Fig 5, Supplementary Fig. S4). Values were over-estimated when SF was specified as lower than its true value (i.e., the clade was larger than thought), and under-estimated when SF was specified as higher than its true value (i.e., the clade was smaller than thought; Supplementary Fig. S4). These effects were most noticeable at lower SFs. Transition rate estimates were similar in correctly specified and mis-specified sets (Supplementary Fig. S4). Top left panel in Figure 4 shows that even though net diversification rate estimates were inaccurate when the SF was mis-specified (i.e., boxplots with wide ranges), particularly in small trees, there was still little overlap between rate estimates for each trait state. This suggests that the model was able to correctly detect which trait state had a comparatively higher net diversification rate and which trait state had a lower rate (i.e., the median for estimates are roughly at the true value).

### Specifying the Sampling Fraction as a Range

When using RevBayes, we found that the signal of state-dependent diversification is correctly recovered in all cases under both sampling methods when the prior distribution of SF was narrow (Table 2). However, when the uncertainty around the true completeness of the dataset is higher and the prior distribution of SF is wider, in 10% of cases the state-independent model was wrongly selected as being the best performing. Even though bayes factor did not point to ETD as the best performing model in those datasets, we note that the rate estimates taken from the MCMC posterior distribution did include the true generating rates. These distributions have an important overlap which is related to the failure to detect state-dependent diversification (Supplementary Fig. S5).

**Table 2:** Number of datasets that were incorrectly (column CTD) and correctly selected (column ETD) during RevBayes analysis using a narrow and wide priors for SF

Prior	Sampling	CTD	ETD
Narrow	Random	0	50
	Bias	0	47
Wide	Random	8	42
	Bias	1	49

Note: The table also includes two different methods of tree tip sampling.

## Discussion

In this study, we have explored how incomplete sampling of phylogenetic trees, and mis-specification of the SF, can affect the ability of SSE models to detect trait dependent diversification and estimate diversification rates. We found that both taxonomic biased sampling and mis-specifying the SF can severely decrease the accuracy of parameter estimation. Sampling fraction mis-specification had more minor effects on model selection, with false positive rates only increasing when the SF was over-specified. Taxonomic biased sampling reduced the accuracy of parameter estimates, more so at lower SFs, and sometimes increased the rates of false positives and false negatives compared to random sampling. When using biased sampled phylogenetic trees, there is a greater risk of obtaining a false positive from a neutrally diverging phylogeny. Smaller phylogenetic trees and any sized phylogenetic trees under low true SFs (< 60%) have higher rates of false positives, rendering them less suitable for current SSE analyses. Although SecSSE was the main method used in this study, our results are relevant to other SSE methods that incorporate hidden traits. The CTD model used is equivalent to the CID-3 model in HISSE (Beaulieu and O’Meara 2016; Herrera-Alsina et al. 2019), meaning our general findings are applicable to HISSE and its relatives.

### Effects of Tree Size

Model selection accuracy was severely reduced in small phylogenies (150–250 tips) even at high SFs (i.e., nearly complete phylogenetic trees). This concurs with previous work using BiSSE, which also showed that small trees (fewer than 300 tips) are less suitable for SSE modeling (Davis et al. 2013). This is because the statistical power of SSE models partially depends on the number of taxa in the phylogenetic tree (Davis et al. 2013). Across all phylogenetic tree sizes, rates of false positives and false negatives were elevated at lower SFs. This is due to decreased sizes of phylogenetic trees and increased uncertainty in the models, both of which come as a consequence of lower SFs.

When interpreting parameter estimates from SSE models, it is therefore important to consider phylogenetic tree size. Interestingly, large phylogenetic trees (1000–5000 tips) under low SF (40%) had similar or better parameter estimates than medium sized phylogenetic trees (450–650 tips) at high SFs (Fig. 4). This suggests that it may be better to use a larger, but incomplete phylogeny, rather than a smaller more complete sub-clade, to study the patterns of trait dependent diversification. The larger (and more complete) the phylogenetic tree, the more accurate the speciation and transition rate estimates.

As in other SSE performance studies (Maddison et al. 2007b; Beaulieu and O’Meara 2016), extinction rate estimates were imprecise, due to the lack of information about extinction in phylogenetic trees (Rabosky 2006). In contrast to some studies (e.g., Höhna et al. 2011), but in agreement with others (e.g., Beaulieu and O’Meara, 2016), we found that some extinction rate estimates tended to be over-estimated. Specifically, the low extinction sets (*µ* = 0.001) tended to have elevated extinction rate estimates. We believe this is due to the extinction rate parameter being set so low, because in the regular extinction rate sets (*µ* = 0.05), extinction rate estimates were generally under-estimated. However, there was a large amount of variation in all parameter estimates. Previous work using BiSSE and simulated phylogenies with 500 tips, showed that speciation rate estimates remain accurate down to ~ 50% SF (Fitzjohn et al. 2009). We concur with this finding and add that speciation rate estimates for larger phylogenetic trees (≥ 1000 tips in the complete in tree) could remain accurate at slightly lower SFs (~ 40%), although accuracy is improved with greater sampling. As suggested by Beaulieu and O’Meara (2016), more accurate net diversification rate estimates can be obtained from larger phylogenetic trees, making it possible to distinguish between trait states with smaller rate differences. However, while larger trees are better suited to SSE analyses in terms of model selection and parameter estimation, the time and computational power required for these analyses is high.

### Tip Ratio Bias and Loss of Trait State Transitions

We found that datasets where the inference analysis failed to select the right model have very similar structure in terms of transition type lost than those datasets where the analysis recovered the right generating underlying process. However, it was often the same phylogenetic trees that had incorrect model selection at different SFs, suggesting that there may be something inherent within these trees that made it more difficult for the model to detect the correct diversification type. The number of trait state transitions lost may have a different impact on simulations with different parameter settings.

Further studies will need to use scenarios with asymmetrical transition rates. Differences in transition rates may be more important when there are trait biases, that is when some trait states are more likely to be sampled than others. Equally, trait biases are more prevalent when transition rates are asymmetrical (Davis et al. 2013). For example, in the scenario where it is easy to transition into a specialist state, but harder to transition out of the specialized state, this asymmetry could lead to trait state biases with more tips in the specialized state, unless this specialized state also had a lower speciation or higher extinction rate. It would be interesting to test how loss of trait state transitions affects SSE models when there are trait biases. Other future work that could be beneficial to further understanding how loss of trait state transitions from incomplete phylogenetic trees affect SSE models include: 1) exploring transitions lost under different transition rate scenarios such as low, medium, and high transition rates and 2) asymmetrical transition rates and different speciation/extinction rates.

### Effects of Sampling Regime

Phylogenetic trees may suffer sampling bias due to certain sub-clades containing greater numbers of rare or undescribed species. In other cases, some species may be deliberately removed from the clade. Overall, if phylogenetic trees are incomplete, our results show that is better for them to be randomly sampled rather than sampled with taxonomic bias. At high SF (80%), biased sampling represents a minor source of inaccuracy: parameter estimates were similar to those from randomly sampled phylogenies, and rates of false positives were lower than in randomly sampled phylogenies. However, when sampling is less complete (SF ≤ 60%), parameter values became over-estimated in biased sampled sets but remained accurate in randomly sampled sets. As SF decreased, rates of false negatives became higher in biased sampled sets compared to randomly sampled sets. This means there is a greater risk of erroneously rejecting trait dependent diversification when a phylogeny is sampled with taxonomic bias.

Similarly to Fitzjohn et al. (2009), we found that randomly trimmed CR generated trees maintained ~ 85% correct model selection across all SFs. In contrast, we found that biased trimmed CR generated trees led to more false positives and to considerable reductions in correct model selection at lower SFs. This means that there is a greater risk of erroneously finding evidence for trait dependent diversification from a biased sampled phylogenetic tree. This may be due to sampling method, as some sub-clades were heavily trimmed (to simulate under-sampling), leading to longer branch lengths, and in the CR model branching patterns are the only information available to estimate diversification.

Currently, the only way to specify the SF in most SSE methods is by trait state, so it is not always possible to account for alternative sampling methods or taxonomic biases. Clade specific SFs were previously enabled in HiSSE but were removed as they caused mathematical errors (Beaulieu 2020); however, clade specific SFs still exist in diversitree (FitzJohn 2012). RevBayes can also account for different sampling methods: uniform (random), diversified, and empirical (clade specific) sampling strategies can be accommodated (https://revbayes.github.io/tutorials/divrate/sampling.html). When it is not possible to sample clades completely, it is recommended to assess the degree of bias in sampling in SSE modeling. When clades are biased sampled and ≤ 60 % complete, extra caution is advised when interpreting the results of SSE models. It is also important to bear in mind that parameter estimates will likely be higher than their true value when trees are biased sampled and have a low SF.

### Effects of Mis-specifying the Sampling Fraction

It can be difficult to accurately set the SF of empirical phylogenetic trees as the actual number of extant species is often unknown. Organisms which may be particularly problematic in this regard include bacteria, archaea, and fungi, as these groups contain many undescribed species (Barns et al. 1994; Lambais et al. 2006; Mueller et al. 2007; Brock et al. 2009; Öpik et al. 2013; Looney et al. 2016). Our findings indicate that it is not acceptable to guess the SF if it is completely unknown: inaccurate SF estimates have a high risk of false positives and inaccurate parameter estimates. Therefore, SSE modeling is most suitable for incomplete reconstructions when the SF is known with some degree of accuracy. Sensitivity analysis to SF specification should be performed to provide confidence to results when the SF has been estimated.

Sampling fraction specifications of ± 20% inaccuracy led to inaccurate parameter estimates. In incompletely sampled phylogenetic trees, the apparent number of speciation and character change events is reduced because tips/branches are missing from the tree. If incomplete sampling is not accounted for, this can lead to likelihoods favoring lower diversification and transition rate estimates (Fitzjohn et al. 2009). When the SF is thought to be higher than it truly is (over-specified; for example, there is a 60% complete phylogenetic tree, but it is specified as 90% complete), not all tips and speciation events will be accounted for, leading to lower speciation rate estimates. Conversely, when the SF is thought to be lower than it truly is (under-specified; for example, there is a 90% complete phylogenetic tree, but it is specified as only 60% complete), the model will account for more tips and speciation events than there actually were, leading to higher diversification rate estimates.

We suggest that parameter estimates are interpreted cautiously when there is uncertainty around the SF approximation. When considering net diversification, the differences in rate estimates with SF mis-specification were only present with biased sampled phylogenetic trees. Even with low levels of sampling, the model correctly detects differences in diversification rates across states so that it tries to maximize the difference between trait 1 and 3 rates. Even though the estimated difference in rates is quite close to the true one (0.4), the overall estimates are inaccurate. This is more evident under bias sampling (Fig. 5, bottom row) where net diversification rate for trait 1 is under-estimated and at the same time, for trait 3 is over-estimated. Interestingly, specifying the correct SF does not lead to better rate estimates under biased sampling. This is likely to be result of 1) confounding extinction with missing branches due to sampling, and 2) SSE models always assume that non-sampled branches are randomly distributed. In the presence of bias sampling, over-specifying SF yields to better estimates. When diversification rate is separated into rates of speciation and extinction, estimates are highly affected by SF mis-specification. However, the relative speciation rate estimates (i.e., which trait states have the lowest and highest speciation rates) is robust to changes in SF specification.

Due to unknown numbers of undescribed species and taxonomic uncertainties, it may be more common for researchers to over-specify the SF, thinking that they have a greater proportion of the phylogeny sampled than they actually do (Vieites et al. 2009; Pimm et al. 2014; Chan et al. 2018; Dickens et al. 2019). Our results show that, in contrast, cautiously under-specifying the SF may not be as bad as over-specifying it: false positive rates were elevated when the SF was over-specified but remained similar to (or even slightly lower than) correctly specified sets when the SF was under-specified. With an 80% complete (randomly trimmed, medium sized) phylogenetic tree, when the SF was correctly specified (as 80%), the rate of false positives was 14.5%; when the SF was under-specified as 60% complete, the false positive rate was slightly lower (13.5%), but when the SF was over-specified as 100% complete, false positives were higher (18%). As SF decreased, rates of false negatives became higher in biased sampled sets compared to randomly sampled sets. This means there is a greater risk of erroneously rejecting trait dependent diversification when a phylogeny is sampled with taxonomic bias. In SSE analyses, we recommend carrying out sensitivity analysis of SF specification that spans, at a minimum ± 20% of the estimated fraction, to determine if the results are robust to variation in the SF specification. The range of SFs used should reflect how much uncertainty there is in the completeness of the tree.

### Bayesian Analysis

One alternative method for dealing with an uncertain SF utilizes Bayesian analyses with a hyperprior placed on the SF. No empirical studies have thus far used this method for dealing with SF uncertainty. More commonly used methods to specify the SF in Bayesian SSE studies are to specify the probability of sampling species within the clade, based on the total number of known species (tips) and the number of species sampled (e.g., Wessinger et al. 2019; Varga et al. 2021), or assuming that all extant species have been included in the phylogeny (e.g., Condamine et al. 2018). Studies using RevBayes and HiSSE that may have benefitted from incorporating uncertain sampling include a study on weevils (Letsch et al. 2018) and a study on basidiomycete fungi (Varga et al. 2021), because these taxa likely have undescribed species. The use of RevBayes in SSE analysis is promising, especially when SF is not well known. However, it is computationally slow which compromises its applicability in large datasets.

### Conclusions and Best Practices

Much progress has been made from the early days of SSE models but work still needs to be done to make SSE methods more robust to the sampling issues explored here. Areas which require further attention include dealing with different types of sampling, uncertainty in tree topology, exploring how loss of trait state transitions affect SSE models, and developing robust methods for confident analyses of smaller phylogenetic trees. Additionally, a more thorough exploration of the efficacy of using a SF prior within a Bayesian framework is needed. Most empirical studies will to some extent violate the assumption that sampling is uniform and random across the phylogeny. It would be highly desirable for SSE methods to be able to account for taxonomic bias. One possibility which could provide more information to the model and decrease uncertainty around the SF, could be to allow for SF specification both by trait state and by clades at the same time; however, this would be challenging to develop (for birth-death process see Höhna et al. 2011).

This work has helped to inform how much error in SF estimates is acceptable, enabling confident SSE modeling of phylogenies where the SF can be reasonably estimated. To conclude, we provide suggestions for best practices when using SSE methods on incompletely sampled phylogenetic trees.

- It may be better to use a larger but somewhat incomplete phylogeny, rather than a smaller but more complete subclade. Larger (≥ 450 tips) and more complete (≥ 60% SF) phylogenetic trees are most suitable for SSE analyses, but tree size is generally more important than completeness.
- Taxonomic biases in sampling can be problematic when phylogenetic trees are < 80% complete. We recommend assessing the degree of bias in sampling, as there are greater risks of false positives and inaccurate parameter estimates when trees are ≤ 60% complete and have been sampled with taxonomic bias. If possible, additional sampling of missing tips is advised, to reduce sampling bias and increase the sampling faction of the phylogeny: increased taxon sampling remains one of the best methods to increase accuracy of inferences drawn from phylogenetic trees (Heath et al. 2008). Inclusion of tips with uncertain trait states is possible in some packages (e.g., SecSSE), and is one possible way to increase the SF.
- For SSE methods, we do not recommend excluding tips from phylogenetic trees, for example because of regional sampling, as this lowers the SF, increases uncertainty in the model, and may increase sampling bias.
- Mis-specification of SF can reduce correct model selection and leads to inaccuracies in parameter estimates. We advise that SSE modeling is most suitable for incomplete phylogenies when the number of extant species in the clade is known with some accuracy. It is worth conducting a thorough examination to estimate the SF as precisely as possible. We suggest two methods for dealing with uncertainty in the SF: 1) using the ML approach, sensitivity analyses should be performed across an appropriate range of SFs (at least ± 20% of the estimated SF), in order to confirm that results are robust to variation in SF specification; 2) using Bayesian analyses of SSE models in order to specify a range of possible SFs as a prior. These methods are not mutually exclusive, and the most confident results may be obtained by implementing both approaches.

## Data Availability

The data underlying this article is available on dryad repository: https://doi.org/10.5061/dryad.wwpzgmsjp.

## Supplementary Material

Data available from the Dryad Digital Repository: https://doi.org/10.5061/dryad.wwpzgmsjp.
